# THE RELATIONSHIP OF DAILY STEPS TO MOOD AND ENERGY: APPLICATION OF STEPMATE (MOBILE APP FOR TRACKING EXERCISE)

**DOI:** 10.1093/geroni/igz038.1922

**Published:** 2019-11-08

**Authors:** Alycia N Bisson, Victoria A Sorrentino, Margie E Lachman

**Affiliations:** Brandeis University, Waltham, Massachusetts, United States

## Abstract

Physical activity is one of the most promising and accessible strategies to promote healthy aging. Yet, the majority of middle-aged and older adults do not engage in the recommended amount of exercise despite awareness of its widespread benefits. Smartphone apps have the potential to be valuable tools in tracking and encouraging physical activity; however, few apps incorporate successful behavior change strategies. Drawing from interviews with older adults, we created a new smartphone app to encourage and track daily walking, StepMATE (Mobile App for Tracking Exercise). StepMATE uses behavior change strategies including action planning and social support to help users determine where, when, and with whom they will walk. The app records steps and also uses experience sampling to assess mood and energy levels twice a day. Adults ages 50 and over (N=58) participated in a one-month study where they used the app to set their own walking goals and track their daily walking. Using multilevel modeling, we found that on days in which adults take more steps than their average, they report higher mood and energy. We also found that on days in which participants achieve their step goal, they report higher mood and energy than on days when they do not achieve their goal. Discussion will center on motivational approaches to behavior change among sedentary older adults.

